# Gait unsteadiness and fall risk in two affective disorders: a preliminary study

**DOI:** 10.1186/1471-244X-4-39

**Published:** 2004-11-24

**Authors:** Jeffrey M Hausdorff, Chung-Kang Peng, Ary L Goldberger, Andrew L Stoll

**Affiliations:** 1 Division on Aging, Harvard Medical School, Boston, MA USA; 2 Laboratory for Gait & Neurodynamics, Tel-Aviv Sourasky Medical Center; Dept. of Physical Therapy, Sackler School of Medicine, Tel-Aviv University, Tel-Aviv Israel; 3 Margret and H.A. Rey Institute for Nonlinear Dynamics in Medicine, Beth Israel Deaconess Medical Center, Harvard Medical School, Boston, MA USA; 4 Psychopharmacology Research Laboratory, McLean Hospital, Harvard Medical School, Belmont MA USA

## Abstract

**Background:**

In older adults, depression has been associated with increased fall risk, but the reasons for this link are not fully clear. Given parallels between major depression and Parkinson's disease, we hypothesized that major depression and related affective disorders would be associated with impairment in the ability to regulate the stride-to-stride fluctuations in gait cycle timing.

**Methods:**

We measured stride-to-stride fluctuations of patients with two forms of mood disorders, unipolar major depressive disorder (MDD) and bipolar disorder, and compared their gait to that of a healthy control group. The primary outcomes were two measures of gait unsteadiness that have been associated with fall risk: stride time variability and swing time variability.

**Results:**

Compared to the control group, the two patient groups tended to walk more slowly and with decreased swing time and increased stride time. However, none of these differences was statistically significant. Compared to the control group, swing time variability was significantly larger in the subjects with bipolar disorder (p < 0.0001) and in the subjects with MDD (p < 0.0004).

**Conclusions:**

Patients with MDD and patients with bipolar disorder display gait unsteadiness. This perturbation in gait may provide a mechanistic link connecting depression and falls. The present findings also suggest the possibility that measurement of variability of gait may provide a readily quantifiable objective approach to monitoring depression and related affective disorders.

## Background

In older adults, depression has been associated with increased fall risk [1,2]. Epidemiological studies indicate that older adults who are more depressed have an increased risk of falls and fractures compared to their aged-matched peers and that this association persists even after adjusting for potential confounding effects such as medication usage [3-5]. The reasons for this link between depression and falls are not fully clear [3]. People with major depression have been shown to walk more slowly, with decreased push-off and more time spent with both feet on the ground [6-8]. These gait changes, however, do not necessarily explain the increased risk of falls associated with depression [9,10]. One possibility is that these two syndromes simply share common risk factors [11]. Alternatively, there may be some cause and effect influences mediating the association between depression and falls. For example, depression may not only lead to a slowed gait, but it could induce gait unsteadiness and a reduction in the ability to maintain a stable walking pattern. Cross-sectional studies suggest that individuals who report more signs of depression tend to be less steady on their feet [12,13]. To date, however, no studies have directly examined the dynamics of gait unsteadiness of patients with major depression or related affective disorders.

Parallels between Parkinson's disease and depression also support the possibility of gait unsteadiness in the latter. Psychomotor studies have revealed deficits in the motor performance of persons with major depression similar to some of the motor disturbances observed in Parkinson's disease [14,15]. In both groups of patients, slowed motor performance can be seen in speech, simple reaction time tests, and gait [14-17]. Furthermore, in both groups, imaging studies reveal shrunken basal ganglia, the motor area primarily affected in Parkinson's disease, and changes in other dopamine dependent neural circuits [15]. In Parkinson's disease, basal ganglia dysfunction not only causes slowed movements, but also contributes to gait unsteadiness and increased stride-to-stride variability [18-20], a marker of fall risk [10,12,20,21].

Given the associations between fall risk and depression and the parallels between major depression and Parkinson's disease, we hypothesized that major depression and related affective disorders would be associated with gait unsteadiness and impairment in the ability to regulate the dynamics of gait stability. To this end, we measured the stride-to-stride fluctuations in the gait cycle timing of patients with two forms of mood disorders, unipolar major depressive disorder (MDD) and bipolar disorder, and compared their gait to that of a healthy control group. The primary outcomes were two measures of gait unsteadiness: stride time variability and swing time variability [12,19,20]. We anticipated that these two variability measures would be increased in the two patient groups compared to the control group. In secondary analyses, we examined the potential confounding influences of age, medications and other subject characteristics on these two measures of variability.

## Methods

### Subjects

Patients with a clinically defined major affective disorder were recruited from the clinics of McLean Hospital, a psychiatric teaching center in Belmont, MA. Specifically, patients were invited to participate if they were being treated for either bipolar disorder or unipolar MDD, as defined by DSM-IV criteria [22]. Patients were excluded if they had clinically significant co-morbidities likely to affect gait including orthopedic disease, dementia, neurological disease, podiatry complaints or any known gait disturbances. To quantify disease severity, we used the Clinical Global Impression Scale (0: healthy; 7 most severe) [23]. Healthy subjects without depression, other affective disorders, or any of the above-mentioned co-morbidities likely to affect gait were recruited from among hospital employees and the community to serve as a healthy control group. The Human Studies committee of McLean Hospital approved the study. All subjects provided informed written consent prior to participating in the study.

### Assessment of gait unsteadiness

Subjects were instructed to walk at their normal pace on level ground for 35 meters, to turn and to walk the same route back, and to continue walking for a total of six minutes. Study subjects were not aware of the specific questions addressed in this investigation.

Previously described methods were used to evaluate gait dynamics and analyze the stride-to-stride fluctuations of gait timing [12,19,20]. Briefly, to measure the gait rhythm and the timing of the gait cycle (i.e., the stride time and the % swing time), force sensitive insoles were placed in the subject's shoes. These inserts produce a measure of the force applied to the ground during ambulation. A small, lightweight (5.5 × 2 × 9 cm; 0.1 kg.) recorder was worn on the ankle and held in place using an ankle wallet. An on board A/D converter (12 bit) sampled the output of the footswitches at 300 Hz and stored the data. Subsequently, the digitized data were transferred to a computer workstation for analysis using software that extracts the initial and end contact time of each stride (i.e., heel-strike and toe-off). With this information, the stride time or duration of the gait cycle (time from initial contact of one foot to subsequent contact of the same foot) and the % swing time were determined for each stride during the walk. The stride time is a measure of the gait cycle duration and the inverse of the cadence. The time spent with one foot in the air, relative to the gait cycle duration, defines the % swing time. Typically, the % swing time becomes smaller in diseases that affect gait (e.g., Parkinson's disease).

To focus on the assessment of the intrinsic dynamics of continuous, usual walking and gait unsteadiness and to insure that the analysis was not influenced by outlier data points, previously described analysis methods were used [12,19,20]. Briefly, the first thirty seconds of each subject's walking time series were excluded to minimize any start-up effects and the last ten seconds were removed as well [12,19,20]. A median filter was then applied to each subject's time series to remove data points that were three standard deviations greater than or less than the median value of each time series [12,13,19]. The mean stride time and mean % swing time were computed. Two measures of stride-to-stride variability [12,19,20] were also assessed. 1) Stride time variability, the magnitude of the stride-to-stride fluctuations in the gait cycle duration, was calculated by determining the coefficient of variation (CV) of each subject's stride time. 2) Swing time variability was determined by calculating the % swing time CV. Values reported are for the left foot. Similar results were obtained if we analyzed each subject's right foot or the "best" foot (i.e., the one with the lowest variability). These two measures of stride-to-stride variability reflect gait unsteadiness and have been shown to be related to fall risk, i.e., larger variability has been associated with increased risk of falls [12,20]. Mean gait speed was evaluated by determining the time required to walk 6 laps (i.e., 210 meters).

### Statistical analysis

Results are reported as means ± standard deviation. For continuous data, the Wilcoxon Rank Sum test (the non-parametric equivalent of the two-sample Student's t-test) was used to compare each patient group to the control group (We note that for all of the primary outcomes the conclusions were unchanged if parametric analyses were applied instead). Subgroup analyses were performed to evaluate the effects of subject characteristics such as age and use of certain drugs. A p-value less than or equal to 0.01 was considered statistically significant (Bonferroni-like adjustment for the five measures of gait). Statistical analysis was performed using SPSS for Windows (version 10.1).

## Results

### Subject characteristics

Fifty subjects (17 women) were studied. Subjects were 36.3 ± 14.7 years old. Twenty-three of the subjects had bipolar disorder (mean age: 43.4 ± 14.2 yrs), nine subjects had MDD (mean age: 40.9 ± 16.9 yrs), and eighteen of the subjects were healthy controls (mean age: 24.9 ± 3.5 yrs). Scores on the Clinical Global Impression (CGI) scale were 0.2 ± 0.4, 3.8 ± 0.9, and 3.2 ± 0.7 for the control, bipolar and MDD groups, respectively. As expected, the mean CGI scores were significantly higher in both patient groups compared to the control group (p < 0.001 for both groups).

### Gait changes in bipolar disorder and MDD

**Figure 1:**
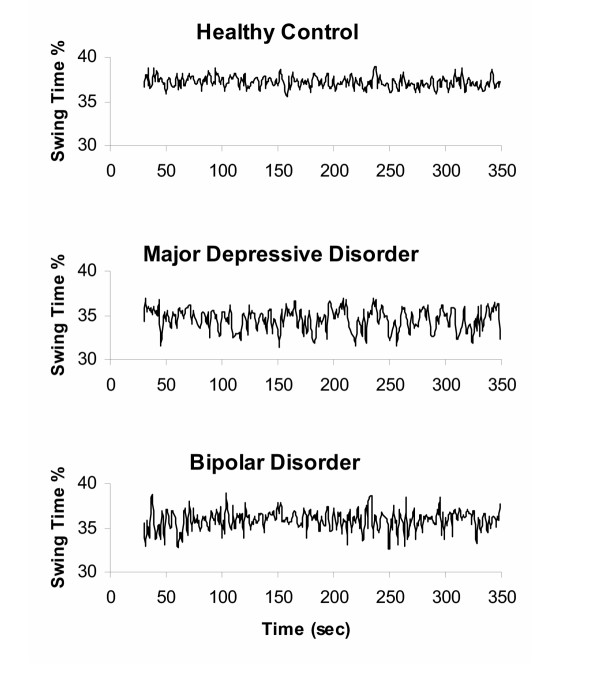
**Representative swing time series from two patients with affective disorders and a control subject. **Data shown are from a healthy control subject (25 yr old male; swing time CV = 1.6 %), a subject with major depressive disorder (38 yr old male; swing time CV = 3.2 %), and a subject with bipolar disorder (20 yr old male; swing time CV = 3.7 %). Note the relatively large stride-to-stride fluctuations (increased variability) in the swing time of the two patients compared with the control subject.

Table 1 summarizes the gait measurements of the three groups. Compared to the control group, the two patient groups tended to walk more slowly, with decreased swing time % and increased stride time. However, none of these differences was statistically significant (e.g., for gait speed, p = 0.32 and 0.10 in patients with bipolar disorder and MDD compared to controls, respectively). As hypothesized, stride time variability tended to be larger in both patient groups compared to the control group, but the differences were not significant (p > 0.29). Compared to the control group, swing time variability was significantly larger in the subjects with bipolar disorder (p < 0.0001) and in the subjects with MDD (p < 0.0004). These measures of gait were not significantly different between the two patient groups. Examples of time series showing the increased stride-to-stride fluctuations in swing time % in a subject with bipolar disorder and a subject with MDD are shown in Figure 1.

**Table 1:** Gait dynamics in the three subject groups

	**Gait Measure**	**Healthy Controls ** **(n = 18)**	**Bipolar Disorder ** **(n = 23)**	**Unipolar Major Depressive Disorder ** **(n = 9)**
Mean Values	Stride time (msec)	1120 ± 69	1161 ± 106	1200 ± 94
	Swing Time (%)	37.0 ± 1.6	35.4 ± 2.1	35.8 ± 1.3
	Gait speed (m/sec)	1.21 ± 0.16	1.14 ± 0.20	1.11 ± 0.14
Variability Measures of Unsteadiness	Stride Time CV (%)	2.4 ± 0.6	2.9 ± 1.3	2.6 ± 0.9
	Swing Time CV (%)	1.9 ± 0.3	3.0 ± 1.4*	3.6 ± 1.9*

CV: coefficient of variation

* p < 0.0005 compared to the control group

All but one of the subjects with MDD was being treated for acute illness, as were nineteen of the subjects with bipolar disorder. Among the subjects with bipolar disorder, the subjects whose disease was stable tended to walk more normally (closer to control values) compared to those subjects with bipolar disorder being treated acutely. Swing time variability tended to be larger (p = 0.044) in the subjects with bipolar disorder who were being treated acutely (3.2 ± 1.5 %) compared to those who were more clinically stable (2.2 ± 0.3 %).

Among the subjects with MDD, swing time variability tended to be higher in subjects on antidepressants (n = 5) compared to subjects not taking antidepressants (p = 0.027). Other measures of gait and the CGI were similar in the MDD subjects who were on or off antidepressants (p > 0.41). One patient with MDD was taking a neuroleptic and none was taking mood stabilizers. Among bipolar patients, there were no significant differences in any of the gait measures in patients who were taking antidepressants (n = 15), neuroleptics (n = 14) or mood stabilizers (n = 12) compared to those who were not. Compared to controls, swing time CV was significantly increased among bipolar patients who were not taking antidepressants (p < 0.001), not taking neuroleptics (p < 0.002) or not taking mood stabilizers (p < 0.001). Stride time CV tended (p = 0.068) to be increased among bipolar patients who were taking antidepressants, neuroleptics, or mood stabilizers (n = 17), compared to those who were not (n = 6). Compared to the controls, swing time CV was significantly increased among the six bipolar patients who were not taking antidepressants, neuroleptics or mood stabilizers (p < 0.007).

Subjects in the two patient groups were generally older than the subjects in the control groups. To evaluate if the group effects were only a result of these age differences, we analyzed the subset of all the subjects who were less than forty years of age. In this subgroup (n = 32), mean ages of all three groups were similar (p > 0.32) and CGI scores remained significantly higher in the two patient groups compared to the control group (p < 0.0001). The results of this subset analysis are consistent with those shown in Table 1. For example, gait speed was similar among the younger subjects with bipolar disorder compared to the younger control subjects (p = 0.185) and among the younger subjects with MDD compared to the younger control subjects (p = 0.075). Among the subjects who were less than forty years old, swing time variability was significantly higher in the subjects with bipolar disorder compared to the control subjects (p = 0.010) and in the subjects with MDD compared to the control subjects (p = 0.002).

## Discussion

Previous studies reported that patients with clinical depression walk more slowly and with less time spent in the swing phase of the gait cycle [6,7]. The present findings lend support to those reports. Although the differences were not statistically significant in this study, subjects with MDD and subjects with bipolar disorder generally tended to walk more slowly and with a shortened swing phase compared to the control group. Here we report, for the first time, changes in the stride-to-stride variability of these two patient groups. In both MDD and bipolar disorder, significant increases in swing time variability were observed. This finding raises the intriguing possibility that dynamic instabilities in the walking pattern may be a heretofore unrecognized feature of gait in severe clinical depression and related affective disorders, including subjects with MDD and bipolar disorder. To our knowledge, this is the first quantitative investigation of gait in bipolar disorder patients.

Although not necessarily apparent to the clinical observer, the present quantitative study suggests that severe clinical depression and related major affective disorders may be associated with an unsteady gait, with certain features reminiscent of impairments in Parkinson's disease. There are, however, subtle but important differences. In advanced Parkinson's disease [18,19], gait speed and swing time are typically reduced to a much greater extent than that seen in the present study (e.g., one study reported a mean gait speed of 1.0 m/sec and a mean % swing time of 33.5 % in subjects with Parkinson's disease [19]; compare with Table 1). In Parkinson's disease, increased stride-to-stride variability is seen in the swing time (as in the present study), but the magnitude of the increase in swing time variability is larger than that seen in MDD and bipolar disorder [19]. For example, a previous study found that the mean stride time variability and swing time variability were 4.4 % and 5.7 %, respectively, among patients with Parkinson's disease [19], values that are much higher than those observed in the present study (compare with Table 1). In addition, whereas in Parkinson's disease increased variability is observed in the stride time as well as in swing time, in MDD and bipolar disorder the gait impairment appears in swing time regulation and the alterations are smaller than those observed in Parkinson's disease.

In some previous studies that have examined both stride time variability and swing time variability, an increase in one measure was accompanied by an increase in the other measure [12,19]. This observation is not surprising since swing time is a key component of gait cycle timing and a sub-phase of the stride time. An interesting question is why increased swing time variability was observed among the patients with MDD and bipolar disease in the present study, while stride time variability was not significantly increased? A number of explanations may be possible. Our findings suggest that in some circumstances swing time variability may be a more sensitive marker of subtle underlying changes in neural control and in the locomotor system's ability to repeatedly and precisely generate and execute neural commands. While stride time variability evaluates consistency of the loading response, as reflected in the variability in the time between consecutive heel-strikes, swing time variability reflects the stride-to-stride consistency of both the loading and unloading of body weight. Thus, when the regulation of gait is largely intact and stride-to-stride variability is relatively small, as in the present study, changes in swing time variability may be present, while stride time variability remains largely unchanged.

Another related explanation for the discrepancy been stride time variability and swing time variability has been suggested by Gabell and Nayack [24]. They proposed that stride time variability reflects gait patterning mechanisms and the rhythmicity of locomotor pacing while variability of stance and swing time more closely mirror dynamic equilibrium and postural control mechanisms. A complete explanation for the difference between swing time variability and stride time variability requires further investigation. We note, however, that such discrepant behavior among different measures of gait variability has also been observed in other studies [25-28]. For example in a study of patients with very mild Parkinson's disease [27], swing time variability was significantly increased while stride time variability was only slightly larger than control values.

In older adults, depression is associated with an increase in the risk of falls and hip fractures [1,3]. The mechanisms responsible for this increase are not clear. The present findings suggest that depression and bipolar disorder may be associated with an increase in stride-to-stride variability which may, in turn, predispose to falls [10,12,20,21]. Young and middle-aged adults who are depressed generally do not have an increased fall risk, probably because depression by itself does not increase stride variability enough to cause falls and because other physiologic systems that help maintain gait and balance stability are generally intact. In older adults, falls are often multifactorial [1]. If age-associated changes occur in multiple systems (e.g., vision, balance), depression may further contribute to fall risk by increasing stride variability and exacerbating instability. One possibility is that the changes in basal ganglia function in depression [15] increase stride variability, as occurs in Parkinson's disease; however, the mechanisms whereby depression and other affective disorders influence gait and impair the regulation of stride variability remain to be determined.

A previous study of gait in depression did not find any significant differences in the gait of subjects who were taking medications compared to those who were not [6]. Specifically, gait speed, stride time and swing time were apparently not related to medication usage. For these specific features of gait, our results support the previous findings. Gait speed and stride time were not related to medication usage in the subjects with bipolar disorder or MDD in the present study. Conversely, while swing time variability was increased relative to control values even in the subgroup of bipolar patients who were not taking any medications, among the patients with bipolar disease and the patients with MDD taking certain medications, variability of gait measures tended to be further increased. Although Lemke et al. did not find a medication effect on gait in patients with depression [6], the suggested relationship between gait and medication usage in the present study in the subjects with MDD is consistent with a recent study which reported that certain antidepressants may impair psychomotor abilities and integrative central nervous system function [29]. However, it is not apparent why such an effect was not observed among patients with bipolar disease and why antidepressants might affect the swing time variability of patients with MDD, but not of patients with bipolar disease.

This preliminary study has a number of limitations. As noted, subjects in the control group were generally younger than the two patient groups (mean differences of 4 and 7 years). However, a number of considerations suggest that the observed increase in swing time variability in the patient groups was not simply a function of aging. 1) Subgroup analysis demonstrates that the differences between the groups persist even when only the relatively young subjects (< 40 years of age) are studied. 2) Like the variability of gait speed and stride length, previous work has shown that the variability of stride time and swing time is similar in healthy young and healthy older adults, and that increases in older adults are a sign of pathology [24-26,30-32]. For example, one study of the effects of physiologic aging on gait found stride time variability was essentially identical in a group of healthy young adults (mean age 24.6 years) and healthy older adults (mean age 75.7 yrs) [31], with values similar to those observed in the present study.

The present study has other limitations and raises additional questions that should be addressed in future studies. Among subjects with MDD, we could not evaluate if there were differences between subjects treated for acute illness and those with more chronic symptoms. Further, among patient with bipolar disorder, we did not control for the patients' acute mood status. The present study may have also been underpowered to detect small differences between the patients with bipolar disorder and MDD. A prospective study of a larger number of patients, perhaps in different age groups before and after they undergo different types of therapy, including pharmacologic interventions, should be helpful in leading to a more complete understanding of the relationships among stride variability, fall risk, and medication usage. Such studies may also be helpful in determining the mechanisms whereby depression and related mood disorders influence stride variability, in assessing how this relationship changes over time and with respect to disease state (i.e., acute or stable symptoms), and in evaluating if stride variability measures may prove clinically useful as an objective measure of major affective disorders. Stride variability is less affected by a conscious effort to alter gait and more closely reflects intrinsic underlying impairment [10]. Additional biomechanical studies may also be helpful in elaborating the mechanisms underlying gait changes in affective disorders and possible causal links between swing time variability, gait instability, and the risk of falls.

## Conclusions

Despite the limitations of this study, the present findings in a relatively small sample size indicate that patients with MDD and patients with bipolar disorder display gait unsteadiness evidenced by increased swing time variability, even while other measures of gait are not markedly changed. This apparent disparity between measures of stride variability, on the one hand, and gait speed and other features of gait, on the other, raises the possibility that stride-to-stride *variability *in major mood disorders may be more sensitive to certain subtle neuropsychiatric influences than measures based only on the *mean *value of a given parameter. Our findings also demonstrate that measurement of variability of gait may provide a readily quantifiable approach to monitoring depression and other affective disorders. Moreover, these results suggest that this perturbation in gait dynamics may provide a mechanistic link connecting depression and falls in older adults.

## List of abbreviations

CV: coefficient of variation

## Competing interests

The author(s) declare that they have no competing interests.

## Authors' contributions

JMH, ALG and ALS conceived of the study, and participated in its design and coordination. JMH drafted the manuscript. JMH and CKP carried out the time series and statistical analyses. All authors helped to revise the manuscript and read and approved the final manuscript.

## Pre-publication history

The pre-publication history for this paper can be accessed here:


